# Trace Element Imbalance and Redox-Relevant Serum Profile in Hemodialysis: A Validated Multielement ICP-MS Study

**DOI:** 10.3390/antiox15040457

**Published:** 2026-04-08

**Authors:** Sara Valderrama-Sanz, Jordi Tortosa-Carreres, Ricardo Molina-Gasset, José-Luis Todolí-Torró

**Affiliations:** 1 Clinical Analysis Service, Hospital Virgen de los Lirios, Polígono de Caramanchel s/n, 03804 Alcoi, Spain; saravalderrama@hotmail.com (S.V.-S.); molina_ric@gva.es (R.M.-G.); 2 Laboratory Department, Hospital Univeritari i Politècnic la Fe, Av Fernando Abril Martorell 106, 46026 València, Spain; tortosa_jorcar@gva.es; 3 Analytical Chemistry Department, Nutrition and Food Sciences, University of Alicante, Carretera San Vicente del Raspeig s/n, 03690 San Vicente del Raspeig, Spain

**Keywords:** hemodialysis, trace elements, oxidative stress, redox imbalance, ICP-MS, chronic kidney disease

## Abstract

Chronic kidney disease (CKD) is associated with persistent inflammation and increased oxidative stress. Trace elements play an important role in these processes as modulators of redox balance, acting either as cofactors of antioxidant systems or as potential inducers of pro-oxidant mechanisms. In this study, the serum profile of trace elements with antioxidant and pro-oxidant relevance was characterized by inductively coupled plasma mass spectrometry (ICP-MS) in hemodialysis patients. Results were compared with those from a healthy control group, and associations with biochemical and hematological parameters were explored. A cross-sectional observational study was conducted including 117 hemodialysis patients and 82 healthy controls, determining a panel of eight trace elements (Zn, Se, Cu, Mn, As, Cr, Co, and Ni). The method showed adequate sensitivity, satisfactory precision for most elements, and acceptable trueness. Hemodialysis patients exhibited significantly lower serum concentrations of Zn and Se, together with greater interindividual variability, as well as higher concentrations of elements with potential pro-oxidant effects, including As, Cr, Co, and Ni. Within this group, Zn and Se were mainly associated with markers of the protein compartment, Cu with ceruloplasmin and inflammatory markers, and Cr and Ni with parameters of renal function and vitamin D status.

## 1. Introduction

Chronic kidney disease (CKD) represents a major global public health challenge, characterized by increasing prevalence and a substantial burden of morbidity and mortality, particularly in its advanced stages [1]. In end-stage renal disease (ESRD), renal replacement therapy is essential, with hemodialysis (HD) remaining the most widely utilized modality [2]. However, despite its efficacy in clearing uremic toxins and maintaining hydroelectrolytic homeostasis, HD patients transition into a complex biological milieu defined by chronic inflammation, metabolic dysfunction, and exacerbated oxidative stress [3,4].

Oxidative stress, defined as the systemic imbalance between the generation of reactive oxygen species (ROS) and the endogenous antioxidant capacity, plays a pivotal role in the progression of advanced CKD. This redox disequilibrium is intrinsically linked to the pathophysiology of several complications, including cardiovascular disease, anemia, malnutrition, immune dysfunction, and increased mortality [5,6]. In the HD population, this state is further aggravated by the retention of pro-oxidant elements, persistent activation of inflammatory pathways, bioincompatibility of the extracorporeal circuit, and the dialytic loss of low-molecular-weight antioxidant molecules [4,7].

Trace elements are critical modulators of this redox balance, acting either as essential cofactors for antioxidant enzymes or as potential initiators of pro-oxidant and inflammatory cascades. Key antioxidant micronutrients include zinc (Zn), which maintains protein structural stability and modulates inflammatory responses as a component of Cu/Zn-superoxide dismutase; selenium (Se), an indispensable constituent of glutathione peroxidases and thioredoxin reductase; and manganese (Mn), a cofactor for mitochondrial Mn-superoxide dismutase. Conversely, copper (Cu), while essential, may exert pro-oxidant effects if its sequestration by transport proteins is compromised. Other elements, such as arsenic (As), chromium (Cr), cobalt (Co), and nickel (Ni), are known to induce ROS generation, mitochondrial dysfunction, and inflammatory activation even at trace concentrations [8,9,10,11,12,13,14].

In HD patients, the homeostasis of these elements is often disrupted by impaired renal excretion, dietary restrictions, altered transport kinetics, and potential exposure during the dialysis procedure itself. Despite previous investigations, many studies suffer from methodological limitations, including small cohorts, lack of healthy controls, or the use of analytical techniques with insufficient sensitivity compared to inductively coupled plasma mass spectrometry (ICP-MS) [15,16,17]. While ICP-MS offers superior multielemental detection, its application to complex biological matrices like human serum necessitates rigorous analytical validation to ensure the reliability of the resulting redox profiles [18,19,20].

Following a robust validation of sensitivity, precision, and trueness, the present study aimed to characterize the serum profiles of Zn, Se, Mn, Cu, As, Cr, Ni, and Co in hemodialysis patients. By comparing these findings with a healthy control group, we explored the associations between these trace elements and key biochemical and hematological parameters to better understand the elemental contribution to the unfavorable redox environment in advanced CKD. In this context, the present study provides a comprehensive multielement characterization using a validated ICP-MS approach, with particular emphasis on interindividual variability and the integration of trace elements within biochemical and hematological association networks.

## 2. Materials and Methods

### 2.1. Study Design and Population

A cross-sectional observational study was conducted including 117 patients undergoing maintenance hemodialysis at Virgen de los Lirios Hospital (Alcoy, Spain) and 82 healthy individuals recruited as controls from the same hospital.

Inclusion criteria for the hemodialysis group were adult age, treatment with chronic hemodialysis, and provision of written informed consent. Patients with active or recent neoplastic disease were excluded because malignancy may affect trace element metabolism and systemic inflammatory status. No additional restrictive criteria were applied in order to obtain a representative population of patients receiving hemodialysis.

The control group consisted of individuals without a known history of kidney disease, with preserved renal function and no active or previous neoplastic disease. Control participants were recruited among individuals attending the hospital for routine blood testing. Controls were selected to obtain a comparable age and sex distribution relative to the hemodialysis group. All control participants provided written informed consent.

Lifestyle-related variables were collected through a structured questionnaire. Regular consumption of meat, fish, salads, and alcohol was defined as intake more than three times per week. Smoking status was recorded as a dichotomous variable (yes/no).

The study was conducted in accordance with the Declaration of Helsinki and was approved by the Ethics Committee of Virgen de los Lirios Hospital (Alcoy, Spain). Written informed consent was obtained from all participants prior to inclusion in the study.

### 2.2. Sample Collection and Preparation

Blood samples were collected following routine clinical procedures. In the control group, blood was obtained by peripheral venipuncture under fasting conditions. Serum was selected as the analytical matrix because it presents fewer matrix-related interferences than whole blood for trace element determination.

Trace element analyses were performed using trace element–free BD Vacutainer^®^ tubes. Serum separator tubes were used for biochemical analyses and K3-EDTA tubes for hematological parameters. In hemodialysis patients, samples were obtained from the vascular access immediately before the dialysis session using the same types of collection tubes.

Samples were centrifuged at 3500 rpm for 7 min. For trace element determination, serum was aliquoted into microtubes previously washed with 1% (*v*/*v*) nitric acid to minimize potential contamination and stored at −80 °C until analysis. Prior to ICP-MS analysis, samples were diluted 1:10 with ultrapure water acidified with 1% (*v*/*v*) nitric acid.

Samples intended for biochemical and hematological analyses were processed immediately according to the routine workflow of the clinical laboratory.

### 2.3. Selection of the Elemental Panel

Eight trace elements were selected based on their relevance to oxidative and antioxidant processes. Zn, Se, Cu, and Mn were considered elements with antioxidant or antioxidant-modulating roles, whereas As, Cr, Co, and Ni were included due to their potential pro-oxidant properties.

### 2.4. ICP-MS Instrumentation and Calibration

Multielement analysis was performed by inductively coupled plasma mass spectrometry (ICP-MS) using an Agilent 7700x instrument (Agilent Technologies, Santa Clara, CA, USA) at the University of Alicante (Spain).

The following isotopes were monitored: ^66^Zn, ^78^Se, ^55^Mn, ^63^Cu, ^75^As, ^52^Cr, ^59^Co, and ^60^Ni. Each sample was analyzed in triplicate under repeatability conditions, and signal variability was assessed through relative standard deviation (RSD).

Signal intensity was expressed as counts per second (CPS), and signal acquisition parameters, including dwell time and integration time, were optimized to ensure adequate sensitivity and precision for all analytes.

Helium collision cell mode was used to minimize polyatomic interferences, particularly for elements such as Cr, Se, and As. Internal standardization (^45^Sc and ^72^Ge) was applied to correct for matrix effects and instrumental drift.

External calibration was performed using multielement standard solutions at multiple concentration levels (0, 0.5, 1, 5, 10, 50, 100, 500, 1000 and 5000 µg L^−1^), covering the expected concentration ranges of the analytes. Calibration curves showed good linearity across the studied concentration ranges for all analytes (R^2^ > 0.99). Instrumental drift and matrix effects were corrected using internal standards (^45^Sc and ^72^Ge).

Table 1 summarizes the ICP-MS operating conditions.

### 2.5. Analytical Method Validation

The analytical method was validated by evaluating limits of detection (LOD), limits of quantification (LOQ), intraday precision (repeatability), intermediate precision (interday precision), and trueness according to EURACHEM recommendations [21].

LOD and LOQ were calculated from the analysis of 30 aqueous blanks using the expressions:

LOD = 3σ/m(1)

LOQ = 10σ/m(2)

where σ represents the standard deviation of the blank and m corresponds to the slope of the calibration curve.

Intraday precision was assessed by performing 30 consecutive measurements of a pooled patient serum sample under repeatability conditions (same instrument, calibration, and operator). Intermediate precision was evaluated by analyzing the same sample on three different days, performing three independent determinations per day (*n* = 9). Precision was expressed as relative standard deviation (RSD, %).

Trueness was evaluated using certified reference control materials (ClinChek^®^ Level I and Level II), performing three replicate measurements on the same day. Results were expressed as percentage recovery relative to the manufacturer-assigned values.

### 2.6. Biochemical and Hematological Analyses

A total of 35 biochemical and hematological parameters were evaluated, including markers of protein and nutritional status, renal function and uremic burden, mineral and vitamin metabolism, lipid profile, inflammatory markers, and hematological parameters.

All analyses were performed at the Clinical Laboratory of Virgen de los Lirios Hospital following standardized procedures and routine internal quality control protocols.

Biochemical parameters were determined using automated spectrophotometric methods on a Cobas 702 analyzer (Roche Diagnostics, Mannheim, Germany). Hormonal and vitamin concentrations were measured using automated electrochemiluminescence immunoassays (Cobas 601 analyzer, Roche Diagnostics, Mannheim, Germany), including parathyroid hormone (PTH), 25-hydroxyvitamin D, vitamin B12, and folate (vitamin B9), whereas ceruloplasmin and prealbumin were determined by immunoturbidimetry (Cobas c311 analyzer, Roche Diagnostics, Mannheim, Germany).

Hematological parameters were analyzed using automated flow cytometry on a Sysmex XE-5000 hematology analyzer (Sysmex Corporation, Kobe, Japan).

### 2.7. Statistical Analysis

Statistical analyses were performed using RStudio (version 2025.05.0, Build 496). Data distribution was evaluated using the Shapiro–Wilk test. Since most variables did not follow a normal distribution, non-parametric methods were applied.

Differences between hemodialysis patients (HD) and controls (CNTRL) were assessed using the Mann–Whitney U test. Multiple testing correction was performed using the Benjamini–Hochberg (BH) procedure.

Values below the limit of detection were treated as left-censored data and conservatively imputed as LOD/2 for statistical analyses.

Associations between serum trace element concentrations and analytical parameters were evaluated separately within each cohort using Spearman’s rank correlation coefficient (ρ). Correlation matrices were generated for each cohort, and *p* values were adjusted for multiple testing using the BH method.

Heatmaps were generated using the ggplot2 package, where color intensity represents the magnitude and direction of correlation coefficients. Statistical significance after correction was indicated using significance symbols.

Data processing and restructuring were performed using the dplyr package, and graphical representations were generated using ggplot2 and patchwork.

## 3. Results

### 3.1. Validation of the Multielement ICP-MS Method

#### 3.1.1. Limits of Detection and Quantification

The limits of detection (LOD) and limits of quantification (LOQ), calculated from 30 aqueous blanks and corrected using the corresponding internal standards, allowed reliable determination of the eight selected trace elements (Zn, Se, Cu, Mn, As, Cr, Co, and Ni) in human serum (Table 2).

For antioxidant-related elements (Zn, Se, Cu, and Mn), LOQ values were below the physiological ranges reported in the general population, ensuring quantification within clinically relevant concentrations. For elements with potential pro-oxidant effects (As, Cr, Co, and Ni), the obtained limits allowed detection of concentrations well below those typically associated with toxicological or clinical relevance.

#### 3.1.2. Precision

Intraday precision was evaluated through 30 consecutive measurements of a pooled patient serum sample under repeatability conditions. Results were expressed as relative standard deviation (RSD, %) (Table 3).

Low intraday variability was observed for Zn, Cu, Se, and As (RSD < 6%). Mn and Co showed intermediate variability (10–20%), whereas Ni and Cr exhibited the highest RSD values (>20%).

Intermediate precision (interday) was assessed by analyzing the same sample on three different days with three independent measurements per day (*n* = 9) (Table 3). Zn, Cu, As, Se, and Mn showed RSD values ≤10%, indicating good reproducibility between days. Co and Ni presented moderate variability (10–20%), whereas Cr showed the highest interday variability, with RSD values close to 30%.

#### 3.1.3. Trueness

Trueness was assessed using certified reference materials (ClinChek^®^ Level I and Level II) (Table 4). Zn, Cu, As, and Co showed recoveries within the commonly accepted range for trace element analysis in biological matrices (80–120%) at both levels.

Se exhibited slightly elevated recovery at the low concentration level and values close to 100% at the high level. Ni and Cr showed recoveries above 120% in the low-level material, while values at the high level were within the acceptable range. For Mn, no certified value was available for Level I; recovery at Level II remained within acceptable limits.

### 3.2. Differences in Trace Element Concentrations Between Controls and Hemodialysis Patients

**Figure 1:**
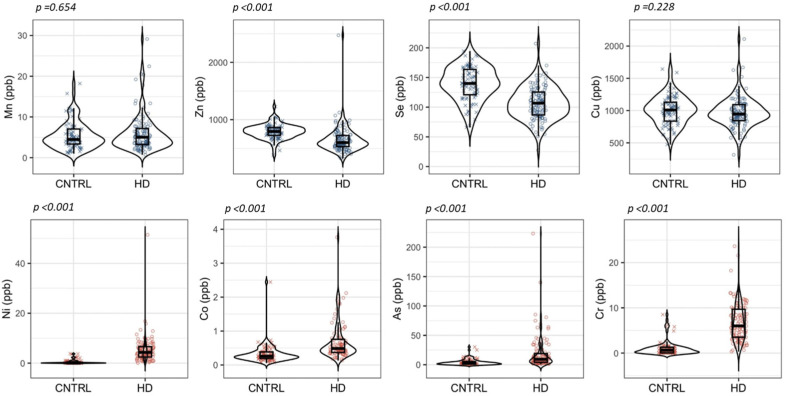
Comparison of serum trace elements concentrations between hemodialysis patients and control subjects. Distribution of serum trace elements concentrations in hemodialysis patients and control subjects, shown as violin plots with overlaid boxplots and individual data points. Group comparisons were performed using the Mann–Whitney U test, and the *p* values displayed correspond to Benjamini–Hochberg-adjusted *p* values. Units are expressed in parts per billion (µg L^−1^). Blue color: antioxidant-related elements; red color: Elements with pro-oxidant potential.

Comparison between healthy controls (*n* = 82) and hemodialysis patients (*n* = 117) revealed a clearly differentiated elemental profile between groups (Table 5; Figure 1). Hemodialysis patients showed lower concentrations of antioxidant-related elements and higher concentrations of elements with potential pro-oxidant properties.

A descriptive comparison of lifestyle-related variables showed no relevant differences between groups in smoking status, alcohol consumption, meat intake, or fish intake, although differences were observed in some dietary habits (Supplementary Table S1).

#### 3.2.1. Antioxidant-Related Elements

Among antioxidant-related elements, Zn and Se showed the most pronounced differences between groups (Table 5; Figure 1).

Serum concentrations of Zn and Se were significantly lower in hemodialysis patients than in controls, and these differences remained significant after multiple comparison correction (*p* < 0.001). In addition to lower median values, both elements displayed increased interindividual variability in the hemodialysis group (Figure 1).

Cu and Mn did not show statistically significant differences after multiple comparison correction (Table 5). However, both elements presented broader distributions and a higher frequency of extreme values in the hemodialysis group (Figure 1).

#### 3.2.2. Elements with Pro-Oxidant Potential

Elements with potential pro-oxidant properties (Ni, Cr, As, and Co) showed an opposite pattern (Table 5; Figure 1).

Serum concentrations of Ni, Cr, As, and Co were significantly higher in hemodialysis patients than in controls (*p* < 0.001 after multiple comparison correction). In addition, the hemodialysis group displayed asymmetric distributions with subgroups showing markedly elevated concentrations, which were largely absent in controls.

#### 3.2.3. Overall Elemental Profile

Overall, hemodialysis patients exhibited a dual elemental profile characterized by decreased concentrations of key antioxidant-related elements (particularly Zn and Se) and increased concentrations of elements with potential pro-oxidant properties (Ni, Cr, As, and Co). These differences involved not only shifts in central tendency but also increased variability in element distributions within the hemodialysis cohort.

### 3.3. Associations Between Trace Elements and Biochemical Parameters

**Figure 2:**
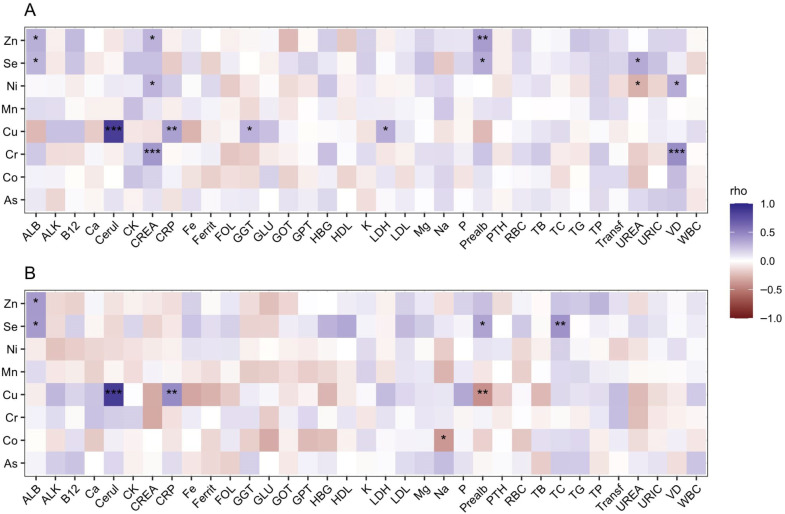
Correlation between serum trace elements and routine laboratory parameters in hemodialysis patients and controls. Heatmaps showing Spearman correlation coefficients (rho) between serum trace elements concentrations and routine laboratory parameters in hemodialysis patients (**A**) and control subjects (**B**). Correlation strength is color-coded according to the scale shown. ALB: albumin; ALK: alkaline phosphatase; As: arsenic; B12: vitamin B12; Ca: calcium; Cerul: Ceruloplasmin; CK: creatine kinase; Co: cobalt; Cr: chromium; CREA: creatinine; CRP: C-reactive protein; Cu: copper; Fe: iron; Ferrit: ferritin; FOL: folate; GGT: gamma-glutamyl transferase; GLU: glucose; GOT: aspartate aminotransferase; GPT: alanine aminotransferase; HBG: hemoglobin; HDL: high-density lipoprotein cholesterol; K: potassium; LDH: lactate dehydrogenase; LDL: low-density lipoprotein cholesterol; Mg: magnesium; Mn: manganese; Na: sodium; Ni: nickel; P: phosphorus; Prealb: Prealbumin; PTH: parathyroid hormone; RBC: red blood cell count; Se: selenium; TB: total bilirubin; TC: total cholesterol; TG: triglycerides; TP: total protein; Transf: transferrin; UREA: urea; URIC: uric acid; VD: vitamin D; WBC: white blood cell count; Zn: zinc. Statistical significance after Benjamini–Hochberg correction is indicated by asterisks (*: *p* < 0.05; **: *p* < 0.01; ***: *p* < 0.001).

Associations between serum trace element concentrations and biochemical and hematological parameters were evaluated using Spearman’s rank correlation separately in hemodialysis patients (HD) and healthy controls (CNTRL). Only associations that remained significant after Benjamini–Hochberg correction are reported (Figure 2).

#### 3.3.1. Hemodialysis Patients

In hemodialysis patients (Figure 2A), a larger number of significant associations were observed compared with controls.

Zn and Se showed consistent positive correlations with albumin and prealbumin, indicating a close relationship with circulating protein status. Additional associations were observed with markers of uremic burden, including correlations between Zn and creatinine and between Se and urea.

Cr and Ni shared correlations with creatinine and vitamin D, linking these elements to renal function and mineral metabolism. Ni also showed a negative association with urea.

Cu displayed the highest number of significant associations, including a strong positive correlation with ceruloplasmin and additional positive correlations with C-reactive protein, gamma-glutamyl transferase (GGT), and lactate dehydrogenase (LDH).

#### 3.3.2. Healthy Controls

In healthy controls (Figure 2B), correlations were fewer and more restricted. Zn showed a single positive correlation with albumin. Se was positively associated with albumin and prealbumin and also correlated with total cholesterol. Cu maintained a strong positive correlation with ceruloplasmin and a positive association with C-reactive protein, while showing a negative correlation with prealbumin. Co presented a single negative correlation with sodium.

#### 3.3.3. Comparison Between Groups

Comparison of the two groups revealed substantial differences in both the number and structure of associations. In healthy controls, correlations were limited and mainly involved variables related to protein transport and metabolic status.

In contrast, hemodialysis patients exhibited a broader association network involving both antioxidant-related elements and elements with potential pro-oxidant properties. These associations extended to parameters related to renal function, uremic burden, mineral metabolism, and inflammatory or enzymatic markers.

## 4. Discussion

The present study provides a comprehensive characterization of the serum trace element profile in patients undergoing hemodialysis using a validated multielement ICP-MS approach. The results reveal a markedly altered elemental pattern in the hemodialysis population, characterized by reduced concentrations of key antioxidant-related elements, particularly Zn and Se, together with increased levels of elements with potential pro-oxidant properties such as Ni, Cr, As, and Co. Importantly, these differences were accompanied by a pronounced increase in interindividual variability and by the emergence of distinct association networks linking trace elements with biochemical and hematological parameters related to protein status, renal function, mineral metabolism, and inflammatory processes. Taken together, these findings suggest that advanced chronic kidney disease and hemodialysis treatment are associated with a substantial disruption of elemental homeostasis that may contribute to the unfavorable redox environment typically observed in this clinical context.

### 4.1. Methodological Aspects of Multielement ICP-MS Analysis

From a methodological perspective, the results confirm the suitability of ICP-MS for the simultaneous determination of trace elements in human serum within a complex clinical context [22]. Although element-dependent variability was observed, the overall analytical performance was consistent with previous ICP-MS studies in biological matrices and allowed reliable comparative evaluation between groups [23,24].

Overall, the method proved adequate for the objectives of the present study, which focused on the characterization of multielement profiles and their associations with clinical parameters rather than on absolute quantification at the individual level.

### 4.2. Elements with Antioxidant Potential

One of the most consistent findings of the present study is the reduction and increased interindividual variability of key antioxidant elements, particularly Zn and Se, in patients undergoing hemodialysis. The decrease in serum Zn, together with its increased dispersion, is consistent with numerous previous studies reporting Zn deficiency in advanced chronic kidney disease (CKD) and dialysis populations, often characterized by marked interindividual variability [13,15,16,17]. These deficiencies have been attributed to multiple factors, including hypoalbuminemia, impaired protein transport, dietary restrictions associated with CKD, alterations in intestinal absorption, and losses related to the dialysis procedure itself [15,16].

The associations observed between Zn and markers of the protein compartment, such as albumin and prealbumin, highlight the strong dependence of serum Zn availability on the nutritional and protein status of hemodialysis patients, a pattern widely described in advanced CKD [13,17,25]. This observation is consistent with studies identifying hypoalbuminemia and malnutrition as major determinants of Zn deficiency in this population [13,26]. Given the structural role of Zn in protein stabilization and its function as an essential cofactor of Cu/Zn-superoxide dismutase, its reduction and dysregulation may indirectly contribute to reduced systemic antioxidant capacity, a phenomenon frequently reported in advanced CKD and hemodialysis patients [7,12].

Similarly, Se showed lower concentrations and increased interindividual variability in hemodialysis patients, a finding consistently reported in previous studies [13,15,16,26,27]. This observation is particularly relevant from a pathophysiological perspective because Se is an essential cofactor of key antioxidant enzymes such as glutathione peroxidases and thioredoxin reductases, which are involved in the neutralization of reactive oxygen species and the maintenance of cellular redox balance [11,13].

The observed associations between Se and albumin and prealbumin further support the notion that circulating Se concentrations are closely linked to the protein compartment, suggesting that its availability largely depends on nutritional status and protein transport mechanisms in these patients. Although the design of the present study does not allow direct evaluation of enzymatic activity, the reduced and more heterogeneous serum Se concentrations observed are compatible with a lower functional availability of this element for Se-dependent antioxidant systems in hemodialysis patients.

Copper exhibited a clearly different pattern compared with Zn and Se. Rather than a uniform deficiency, Cu showed marked interindividual dispersion, with elevated values observed in a subgroup of patients, a pattern previously described in hemodialysis populations [28]. This heterogeneity is consistent with the dual nature of Cu, which is essential for antioxidant defense but may exert pro-oxidant effects when its homeostasis is disrupted [29]. In the present study, the strong correlation between Cu and ceruloplasmin confirms their structural relationship, whereas its associations with C-reactive protein, GGT, and LDH place Cu within a biochemical environment linked to systemic inflammation and oxidative stress. These findings suggest that total serum Cu may partly reflect persistent inflammatory processes characteristic of hemodialysis rather than a protective antioxidant state.

Manganese, although present at very low concentrations, also showed greater heterogeneity in hemodialysis patients but did not display statistically significant associations with the evaluated biochemical and hematological parameters. Given its role as a cofactor of mitochondrial Mn-superoxide dismutase [14], this variability could reflect subtle alterations in the regulation of this enzyme, although the present results do not allow firm functional conclusions beyond the observed increase in interindividual variability.

### 4.3. Elements with Pro-Oxidant Potential

Another relevant finding of this study is the difference in serum concentrations of elements with potential pro-oxidant properties between hemodialysis patients and healthy controls. This group includes As, Co, Cr, and Ni, which showed higher concentrations in the hemodialysis population.

Similar observations have been reported previously in patients with advanced CKD and in dialysis populations, where heterogeneous alterations in potentially toxic trace elements have been described. These alterations are typically characterized by high interindividual variability and by inconsistent findings across studies, reflecting the influence of multiple factors including residual renal function, dialysis modality, environmental exposure, and analytical methodology [15,16,17,26,27].

Within this group, Cr and Ni were particularly notable because they also showed specific associations with biochemical parameters that were absent in healthy controls. Both elements shared significant correlations with creatinine and vitamin D, integrating them into a common axis related to renal function and mineral metabolism. Previous studies have described increased accumulation of Cr and Ni in hemodialysis patients and associations with markers of renal function, suggesting that reduced renal excretion and metabolic alterations characteristic of advanced CKD influence their serum behavior [6,13,16,17]. In addition, the negative association between Ni and urea observed in this study further highlights the complexity of its regulation in the uremic environment and is consistent with the heterogeneity described in the literature for this element [6,16].

These findings reinforce the concept that the behavior of elements with potential pro-oxidant properties in hemodialysis cannot be interpreted solely in terms of absolute concentration. Rather, their pathophysiological relevance also depends on their integration within the altered biochemical environment of uremia, which varies between elements and between individuals [10,15,16].

### 4.4. Pathophysiological Implications of Redox Imbalance in Hemodialysis Patients

The imbalance observed between elemental antioxidant support and the reorganization of elements with potential pro-oxidant properties in hemodialysis patients has relevant pathophysiological implications within an already complex clinical setting. End-stage renal disease is characterized by a high inflammatory burden and systemic metabolic dysfunction, processes closely modulated by redox status [5,6,7].

The reduction in antioxidant support, mainly reflected by the alterations in Zn and Se observed in this study, may compromise the organism’s capacity to neutralize reactive oxygen species. Given the role of these elements as cofactors of key antioxidant systems—including superoxide dismutases and glutathione peroxidases—their reduction and dysregulation are associated with increased susceptibility to oxidative damage at cellular and tissue levels [10,11,12,13]. In hemodialysis patients, this phenomenon may contribute to the persistence of the unfavorable redox environment typically observed in advanced CKD [7,30].

In parallel, the greater biochemical integration of elements with potential pro-oxidant effects, such as Cr and Ni, within metabolic axes related to renal function may promote the generation of reactive species and the activation of redox-sensitive signaling pathways [10,31,32]. Such pro-oxidant conditions have been widely implicated in endothelial dysfunction, mitochondrial impairment, and sustained activation of inflammatory responses, all of which play important roles in the pathophysiology of hemodialysis [7,30].

Taken together, these findings support the concept that redox imbalance in hemodialysis is not an isolated phenomenon but rather a cross-cutting component of the uremic environment that may contribute to the overall pathophysiological complexity of these patients. From this perspective, characterization of the elemental profile provides a useful framework for understanding how alterations in redox status may influence clinical outcomes in end-stage renal disease.

Beyond previously reported alterations in trace element concentrations, the present study provides an integrated view of multielement behavior, including variability patterns and association networks, which may contribute to a more comprehensive understanding of redox-related alterations in hemodialysis patients.

### 4.5. Limitations

The present study has several limitations that should be considered when interpreting the results. First, the analysis is based on total serum concentrations of trace elements, without information on their chemical speciation or on the biologically active fraction. This aspect is particularly relevant for elements such as As, Cr, or Cu, whose physiological impact depends strongly on their chemical form and compartmentalization. Some potentially relevant toxic elements, such as Cd and Pb, were not included in the analytical panel, as the study focused on trace elements that could be reliably quantified in serum under validated conditions.

Second, the study did not directly evaluate the functional activity of antioxidant systems dependent on these elements, such as glutathione peroxidases or superoxide dismutases, which limits the ability to establish a direct relationship between the observed elemental alterations and effective antioxidant capacity.

Third, lifestyle-related variables were collected through a structured questionnaire; however, these variables were assessed in a simplified manner and were not designed to support a detailed nutritional analysis. Therefore, their potential influence on redox status cannot be completely excluded. In addition, participants were recruited from the same geographical area, which may have reduced variability in environmental exposure between groups.

Finally, the cross-sectional design limits causal interpretation of the observed associations and does not allow evaluation of the temporal evolution of the elemental profile or its relationship with disease progression. Longitudinal and mechanistic studies will therefore be necessary to further clarify the functional role of trace elements in advanced CKD and in hemodialysis patients.

## 5. Conclusions

In conclusion, this study demonstrates that hemodialysis is associated with a complex and heterogeneous alteration of the serum trace element profile, affecting both elements with antioxidant functions and those with potential pro-oxidant properties. The reduction and increased interindividual variability of Zn and Se, together with the differential accumulation of certain pro-oxidant elements, reflect a biochemical environment consistent with the unfavorable redox state characteristic of end-stage renal disease.

Network-based association analysis further indicates that, beyond absolute concentrations, the pathophysiological relevance of trace elements in hemodialysis depends on their integration within the metabolic and uremic environment, particularly in relation to nutritional status and renal function. The validated multielement ICP-MS approach enables the identification of co-variation patterns that are not evident in univariate analyses, providing a more comprehensive view of the redox environment in these patients.

Overall, these findings highlight the relevance of trace elements as modulators of redox balance in hemodialysis and provide a solid basis for future research aimed at clarifying their functional role and their potential value as biomarkers or targets for monitoring and therapeutic intervention in advanced chronic kidney disease.

## Figures and Tables

**Table 1:** ICP-MS operating conditions.

Plasma		
Ar flow rates/L min^−1^	Plasma gas: 15.0 Auxiliary gas: 0.9	
RF power/W	1600	
Sampling depth/mm	10	
Liquid aspiration conditions	350 rpm (air)	25 µL min^−1^
Nebulization flow rate/L min^−1^	0.4	
Number of replicates	3	
Integration time/s	0.1	0.3
Sweeps per replicate	1	100
Collision cell		
He flow rate/mL min^−1^	4.3	
HMI System Conditions		
Ar HMI flow rate/L min^−1^	0.56	

**Table 2:** Limits of detection (LOD) and quantification (LOQ) for serum trace elements determined by ICP-MS.

Element	LOD (µg L^−1^)	LOQ (µg L^−1^)
Zn	0.151	0.50
Se	0.216	0.71
Mn	0.072	0.24
Cu	0.160	0.53
As	0.022	0.07
Co	0.017	0.06
Ni	0.041	0.14
Cr	0.037	0.12

**Table 3:** Intraday and interday precision of the ICP-MS method for serum trace element determination.

	Intraday Precision			Interday Precision		
Element	Mean	SD	RSD%	Mean	SD	RSD%
Zn	1074	55	5.2	925	16	1.7
Se	186	4.8	2.6	152	9.8	6.4
Mn	3.5	0.7	18	2.4	0.2	9.5
Cu	1252	20	1.6	1194	30	2.5
As	7.3	0.3	4.1	4.0	0.2	5.4
Co	0.6	0.1	14	0.3	0.06	19
Ni	1.8	0.4	26	1.9	0.3	15
Cr	0.9	0.2	25	2.3	0.7	30

**Table 4:** Trueness evaluation of the ICP-MS method using certified reference materials (ClinChek^®^ Level I and Level II).

	ClinChek^®^ Level I			ClinChek^®^ Level II		
Element	Mean	Cert. I	Recovery (%)	Mean	Cert. II	Recovery (%)
Zn	1224	1320	93	1781	2040	87
Se	84	66	127	1601	105	102
Mn	4.3	-	-	10	9.4	109
Cu	734	801	92	1224	1340	91
As	11	9.9	114	23	19.4	117
Co	2.1	1.7	119	3.3	3.1	108
Ni	5.2	4.0	129	7.5	8.8	85
Cr	5.6	3.9	144	8.5	8.0	106

**Table 5:** Comparison of serum trace element concentrations (µg L^−1^) between hemodialysis patients and control subjects ^1^.

Element	Median (IQR) HD	Median (IQR) CNTRL	p_raw_	p_adj_
Zn	600.2 (530.5–724.1)	794.8 (725.3–861.1)	<0.001	<0.001
Se	106.90 (86.90–125.5)	140 (120.9–163.7)	<0.001	<0.001
Mn	5.01 (3.24–7.16)	4.48 (3.35–7.03)	0.65	0.65
Cu	947.2 (845.2–1093)	1009 (837.5–1130)	0.20	0.23
As	9.37 (4.11–19.10)	2.98 (1.04–5.42)	<0.001	<0.001
Co	0.44 (0.29–0.66)	0.26 (0.19–0.38)	<0.001	<0.001
Ni	4.30 (2.45–6.63)	0.04 (0.04–0.50)	<0.001	<0.001
Cr	6.00 (3.51–9.68)	0.65 (0.04–1.30)	<0.001	<0.001

^1^ Values are reported as median (interquartile range). Group comparisons were performed using the Mann–Whitney U test, with Benjamini–Hochberg correction applied for multiple comparisons. Values below the limit of detection (LOD) were imputed as LOD/2 for statistical analyses. CNTRL: control subjects; HD: hemodialysis.

## Data Availability

The data presented in this study are available from the corresponding author upon reasonable request. The data are not publicly available due to privacy and ethical restrictions.

## Supplementary Materials

The following supporting information can be downloaded at: https://www.mdpi.com/article/10.3390/antiox15040457/s1, Table S1. Lifestyle-related variables in hemodialysis patients and controls.
