# Supplementary material for: Trace Element Imbalance and Redox-Relevant Serum Profile in Hemodialysis: A Validated Multielement ICP-MS Study
Source: Antioxidants (Basel). 2026 Apr 8;15(4):457. doi: 10.3390/antiox15040457 (PMC13113736; doi:10.3390/antiox15040457)
Supplement: Supplementary file 1 [file antioxidants-15-00457-s001.zip › antioxidants-4213503-supplementary.pdf]

Table S1. Lifestyle-related variables in hemodialysis patients and controls.

Variable	Hemodialysis (n=117)	Controls (n=82)
Smoking	18 (15.4%)	11 (13.4%)
Alcohol	22 (18.8%)	19 (23.2%)
Meat intake	68 (58.1%)	49 (59.8%)
Fish intake	28 (23.9%)	22 (26.8%)
Salad intake	36 (30.8%)	58 (70.7%)

Data are presented as number of individuals (percentage). Regular consumption of meat, fish, salads, and alcohol was defined as intake more than three times per week. Smoking status was recorded as yes/no.
